# Knowledge of Pre-Pregnancy Care Among Men Attending the Outpatient Clinics of Hospital Universiti Sains Malaysia

**DOI:** 10.21315/mjms2021.28.2.11

**Published:** 2021-04-21

**Authors:** Siti Hartini Ishak, Lili Husniati Yaacob, Azlina Ishak

**Affiliations:** Department of Family Medicine, Universiti Sains Malaysia, School of Medical Sciences, Universiti Sains Malaysia, Kubang Kerian, Kelantan, Malaysia

**Keywords:** preconception care, men, knowledge

## Abstract

**Background:**

Men’s involvement in pre-pregnancy care is important to ensure a positive pregnancy outcome. The objective of this study is to determine the level of knowledge of pre-pregnancy care among men and the factors associated with poor knowledge.

**Methods:**

This work is a cross-sectional study conducted at the outpatient clinics of Hospital Universiti Sains Malaysia involving 235 married men. A self-administered questionnaire was used and it consisted of four sections: socio-demographic data, reproductive characteristics of couples, clinical characteristics and knowledge of pre-pregnancy care.

**Results:**

More than half of the men (51.9%) had poor knowledge of pre-pregnancy care, mostly on high-risk pregnancy, consequences of poor birth spacing and effect of maternal anaemia on a baby. The mean (SD) knowledge was 11.86 (3.85). Poor knowledge of pre-pregnancy care was significantly associated with age (adjusted odds ratio [AOR] = 0.96; 95% CI: 0.94, 0.99, *P* = 0.002) and education level (AOR = 2.61; 95% CI: 1.49, 4.57; *P* = 0.001).

**Conclusion:**

The men in our study had poor knowledge of pre-pregnancy care. Further health promotion and education are needed to be focused on men to increase their knowledge and share the responsibilities in maternal health.

## Introduction

Pre-pregnancy care is essential because it lays the foundation for the future health of the mother, her child and her family. It includes health promotion, risk factor identification and management and family planning for birth spacing before women proceed with the pregnancy. Its goal is to optimise the potential for a healthy pregnancy and baby in future pregnancies (1). Therefore, mortality and morbidity among women and children can be prevented.

Men, being the leader of the family, are the primary decision-makers of most families in developing countries (2). Their involvement in decision-making on pre-pregnancy care among couples may provide an important strategy in achieving women’s empowerment, which will ultimately result in reduced maternal and neonatal morbidity and mortality (3–4). Men’s involvement has been shown to improve maternal healthcare utilisation in many aspects, such as the number of visits to the antenatal clinic (ANC), ANC visits within the first 12 weeks of pregnancy, HIV testing and birth in a health facility (5). However, globally, the involvement of men in maternal healthcare is low (6) and has not improved much, especially in the Asian region in the past decade (7–8).

Several studies have shown that only around 50% of men knew that certain factors, such as family health history, cigarettes, type of diet intake and folic acid supplement and age, could have serious consequences on the outcome of pregnancies (9–11). Improving men’s knowledge of pre-pregnancy care can improve pregnancy outcomes, ensuring all pregnancies are planned and wanted and enhancing the health of female partners (12). Failure to incorporate men in the maternal health promotion, prevention and care programmes by policymakers and implementers of maternal health services can seriously affect the health of women and the success of these programmes.

Studies have shown that age, education level, family history of chronic disorder, degree of consanguineous marriage, income and number of children are associated with men’s knowledge of pre-pregnancy care (8–9, 13–16). Some factors influencing knowledge about preconception care are also associated with the positive involvement of men in the area (8, 13).

Currently, there are still limited data on the knowledge of pre-pregnancy care among men, especially in the Asian region. The objectives of this study are to determine men’s knowledge of pre-pregnancy care and the factors associated with that knowledge. The results of this study are hoped to shed light on solutions to improve men’s involvement in pre-pregnancy care, which can indirectly enhance overall maternal and neonatal health.

## Methods

This work is a cross-sectional study conducted among men attending the outpatient clinics of Hospital Universiti Sains Malaysia (Hospital USM). Hospital USM is a tertiary hospital and a referral centre located in Kelantan, which is in the east coast part of Malaysia.

### Sample Size Calculation

The sample size was calculated using a two-proportion formula, with the level of men’s education value of 0.64, based on Ampt et al. (8). The calculated sample size after considering a 10% dropout rate was 235 participants.

### Data Collection

Married men aged more than 18 years old and less than 65 years old who were literate were approached to join the study using convenience sampling. Those who consented to participate were given a self-administered questionnaire to answer.

### Instruments

This study used a self-administered questionnaire with four sections adapted from the study of Rosnah and Aisyah (17) conducted in Semenyih, Selangor, on the awareness of pre-pregnancy care in women. The questionnaire was chosen because it was in the Malay language and was suitable for a community survey. Furthermore, the questionnaire was validated, with a Cronbach’s alpha of 0.79. A pilot study was conducted involving 30 men to test for face and content validity among male respondents.

The first section of the questionnaire consisted of the participants’ sociodemographic data: age, occupation, race, religion, education level and income. Data on the wife were also included, such as age, occupation and education level. The second section consisted of the reproductive characteristics of the couples: number of children, age of the youngest child and pregnancy plan. The third section presented the clinical characteristics, such as the presence of a chronic medical illness of the wife.

The final section consisted of the knowledge on pre-pregnancy care and covered seven domains: i) high-risk pregnancy (4 items); ii) effect of poor spacing (4 items); iii) birth spacing (3 items); iv) healthy diet for pregnant women (4 items); v) harmful effect of smoke exposure on babies (1 item); vi) folic acid supplementation before planned pregnancy (1 item) and vii) effect of maternal anaemia on babies (4 items). A correct response for each question was scored as 1, and an incorrect or a ‘don’t know’ response was scored as 0. The total scores were obtained by adding the response scores, with a minimum possible score of 0 and a maximum score of 21. Categorical responses (good/poor) were then generated for the knowledge. Grading was determined according to the mean score from Rosnah: ≤ 12 indicates poor knowledge and > 12 indicates good knowledge (17).

### Statistical Analysis

Descriptive statistics were used to analyse the socio-demographic and knowledge scores, and the results were presented as means and frequencies. The association between the poor knowledge of pre-pregnancy care among men and the sociodemographic and clinical factors was analysed using a simple and multiple logistic regression. The significance level was set to a *P*-value < 0.05, with a 95% confidence interval (CI). All analyses were performed using the Statistical Package for Social Sciences SPSS version 24.

## Results

A total of 235 participants were recruited for the present study, and the response rate was 100%. Table 1 describes the characteristics of all the participants. The majority were Malay, and the mean age of the respondent was 42.1 (SD 11.12) years old. Most of them had a secondary school education and were employed. The majority of the participants did not plan for pregnancy.

The mean knowledge score was 11.8 (SD 3.85). Among the respondents, 122 (51.9%) had poor knowledge of pre-pregnancy care and 113 (48.1%) had good knowledge of pre-pregnancy care.

Table 2 shows the correct responses for knowledge of pre-pregnancy care. It also shows the inadequate knowledge of pre-pregnancy care among the respondents in several aspects of pre-pregnancy care. The majority of the respondents were not aware of the factors that could put a pregnancy at high risk, for example, maternal age of less than 18 years old. Moreover, most of the respondents did not know the effect of maternal anaemia on the unborn baby. By contrast, the participants’ knowledge of good birth spacing was good, as most of them were able to identify the correct answer.

A simple logistic regression showed a significant association between poor knowledge and age of respondent, age of wife and education level (Table 3). A multiple logistic regression showed that age of respondent and education were significantly associated with poor knowledge of pre-pregnancy care (Table 4). No interaction among the factors was found, and no multicollinearity was observed. For each year increase in age, there was a 4% decrease in odds of having poor knowledge (95% CI: 0.94, 0.99; *P* = 0.002). Primary and secondary education had 2.6 times higher odds of having poor knowledge than tertiary education when adjusted for age (95% CI: 1.49, 4.57; *P* = 0.001).

## Discussion

In our study, more than half of the participants (51.9%) had poor knowledge of pre-pregnancy care, with a mean knowledge score of 11.8 (SD 3.85). To our knowledge, no study has been conducted on the knowledge level of pre-pregnancy care among men in Malaysia, and therefore a comparison with similar studies could not be made. However, a similar study on pregnant women conducted in Malaysia in 2015 using the same questionnaire showed similar findings, in which the mean knowledge score was 11.37 (SD 3.94) and 49.1% of the respondents had poor knowledge of pre-conception care (18). The level of knowledge of preconception care among men and women was almost similar.

In general, knowledge of the participants about the factors that put a pregnancy at high risk was inadequate. Most of the participants did not know that becoming pregnant at age 18 years old or below was considered a high-risk pregnancy. This is concerning, as it has been reported that the adolescent fertility rate in Malaysia is 6 births per 1,000 in women aged 15–19 years old (19). Outreach and awareness must begin among adolescents of both genders to improve the health of women and new-borns and to reduce the rates of prematurity and low birth weight associated with teenage pregnancy.

Conversely, most of the participants knew that pregnancy in women over 35 years old is associated with a higher risk. This finding is consistent with a study on Canadian men and women about perinatal risks, with half of the respondents (men and women) recognising that women over the age of 35 years old are more likely to experience difficulty conceiving, to have a baby with Down Syndrome and to develop gestational diabetes (11).

Knowledge of the effect of maternal anaemia was another aspect that was inadequate in our respondents. Our study revealed that men’s knowledge of the effect of maternal anaemia on a baby was poor, as most of them were not aware that maternal anaemia could negatively affect a baby’s health. This is worrying given the fact that the prevalence of anaemia among pregnant women in Malaysia is around 35% (20). However, a local study on women showed that women’s knowledge was slightly better, with 67.4% correctly identifying maternal anaemia as a risk factor for low birth weight (18). Anaemia is a condition that can be prevented and managed through proper nutrition and appropriate birth spacing even before pregnancy is attempted. Therefore, adequate knowledge of both parents in this area is important.

Knowledge on birth spacing is required for potential parents to plan for pregnancy. Planning for pregnancy is important, as couples can optimise not only their living conditions but also their health before conception. This was shown in a study on men attending antenatal check-ups with their partners in London. In this study, 74% of the pregnancies were planned and male ‘planners’ were more likely than other men to take action to improve their health by reducing smoking, reducing alcohol consumption and eating more healthily in preparation for pregnancy (21). Although most of the participants answered correctly in good birth spacing, this was not reflected in their practice in terms of planning for pregnancy, as more than 60% did not plan their pregnancy. This finding is similar to a local study in which the prevalence of family planning practice was 38.7%, and only a third of the male respondents strongly agreed that pregnancy should be planned and discussed together between husband and wife (22). The low figure for planned pregnancy in this study correlates with another study conducted on females in this country (18). This is in contrast with a recent study in Sweden, which found that 81% of pregnancies were planned (23). A possible reason for the difference in the findings is the education level of the participants. The participants from the Swedish study were mainly educated, with 75% of them having a degree.

Although the present study did not look into the association between knowledge and practice, it showed that education level is a significant factor associated with knowledge of pre-pregnancy care. Men with primary and secondary education were three times more likely to have poor knowledge than those with tertiary education. Level of education not only influenced the level of knowledge but also the practice of pre-pregnancy care, as shown across many previous studies (8, 13). A study conducted among married men regarding their role in family planning had a similar finding. The author reported that men with a tertiary education level were three times more likely to discuss the family planning method (13). A study conducted in Myanmar revealed that men with high school or university education were more likely to demonstrate higher levels of involvement in maternal health (8). The same finding was shown in a study conducted among females in Kedah: women who had a higher level of education were four times more likely to use pre-pregnancy care services (24).

Another factor which was associated with knowledge is age. This study found that those with increasing age had lower odds of having poor knowledge. This finding is supported by another study conducted among males in a younger age group: the participants had low knowledge of pre-pregnancy care. This study was conducted among undergraduate students to examine their awareness of issues related to preconception health and pregnancy, with the majority of the participants’ age (98%) 18–24 years old. The male undergraduate students were found to have poor awareness of folic acid, prenatal development, health and pregnancy spacing. They also demonstrated a moderate level of awareness of the importance of preconception care (14). A possible explanation for this is that older men have more firsthand experience with their partners’ previous pregnancies, thus increasing their knowledge of pre-pregnancy care. The finding that nearly two-thirds of our participants have children aged up to five years old supports this explanation.

Although this study did not examine the effect of men’s age on pre-pregnancy practice, multiple studies have shown an association between the two factors. A local study on the involvement of men in family planning found that older age is one of the factors associated with a higher likelihood of men discussing family planning activities (13). Several studies on women have also found that older age is associated with more knowledge of pre-pregnancy care. A study in Ethiopia reported that women aged 25–34 years old were more than two times more knowledgeable about preconception care than those aged 15–24 years old. Women in the age group of 35–49 years old were four times more likely to have better knowledge about preconception care than those who were in the age group of 15–24 years old (25). This higher level of knowledge has been shown to affect the practice of preconception care. A local study on pre-pregnancy care service usage among women of reproductive age in Kedah, Malaysia, found that women aged 35 years old or older were more likely to use pre-pregnancy care services (24). Oza-Frank et al. (26) reported a similar finding: women aged ≤ 19 years old had decreased odds of using preconception care compared with women aged ≥ 35 years old.

This study has several limitations. The study was conducted in a Malay-dominant community and thus a generalisation to other racial groups cannot be made. An assessment of the knowledge of contraception and its use should have been included in the questions, as contraception is a major factor in preconception care. Despite these limitations, the findings can be used as a basis to understand how health practitioners can improve men’s participation in preconception care in Malaysia.

## Conclusion

The knowledge level on pre-pregnancy care of the men in this study was low, as more than half had poor knowledge of pre-pregnancy care. Age and education level were found to be significantly associated with poor knowledge among men. A more concerted educational programme on preconception care should be directed to men to improve their knowledge and ultimately their participation in preconception care.

## Figures and Tables

**Table 1:** Socio-demographic characteristic of respondents (*n* = 235)

Variables	Mean (SD)	*n*	(%)
Age (year)	42.1 (11.12)		
Age of wife (year)	38.4 (10.35)		
Education			
Primary and secondary		132	(56.2)
Tertiary		103	(43.8)
Wife’s education			
Primary and secondary		133	(56.6)
Tertiary		102	(43.4)
Employment			
Employed		229	(97.4)
Unemployed		6	(2.6)
Wife’s employment			
Employed		90	(38.3)
Unemployed		145	(61.7)
Income			
< RM1000		57	(24.3)
RM1001–2000		65	(27.7)
RM2001–3000		47	(20)
> RM3001		66	(28.1)
Number of children			
1–2		77	(32.8)
3–4		113	(48.1)
> 5		45	(19.1)
Age of youngest child			
Less than 1 year old		60	(25.5)
Between 2 and 5 years old		78	(33.2)
More than 5 years old		97	(41.3)
Planned pregnancy			
Planned		85	(36.2)
Unplanned		150	(63.8)
Wife’s chronic medical illness			
Yes		60	(25.5)
No		175	(74.5)

**Table 2:** Correct responses for knowledge on pre-pregnancy care

Item	*n*	(%)
High risks during pregnancy		
i) Mother below 18 years old	89	(37.9)
ii) Mother with small body size	92	(39.1)
iii) Mother pregnant with first child at the age of 35 years old and above	135	(55.3)
iv) Mother with twin pregnancy	104	(44.4)
Poor spacing can cause		
i) Anaemia	107	(45.5)
ii) Birth defect	82	(34.9)
iii) Premature birth	103	(43.8)
iv) Postpartum haemorrhage	106	(45.1)
Recommendation for good birth spacing practices		
i) 1 year old	177	(75.3)
ii) Between 2 and 4 years old	202	(86.0)
iii) More than 5 years old	174	(74.0)
Pregnant mother should		
i) Eat a balanced diet more often than non-pregnant women	213	(90.6)
ii) Eat more iron rich food	202	(86.0)
iii) Eat more calcium rich food	207	(88.1)
iv) Eat less fatty food	187	(79.6)
Exposure to cigarette smoke can harm baby	229	(97.4)
Women planning to get pregnant must take folic acid supplement to prevent birth defect	138	(58.7)
Mother with anaemia can cause baby to		
i) Have low birth weight	129	(54.9)
ii) Look pale	14	(6.0)
iii) Have increased appetite	55	(23.4)
iv) High blood pressure	46	(19.6)

**Table 3:** Associated factor for poor knowledge level by simple logistic regression

Variable	Crude OR	(95% CI)	*P*-value
Age	0.97	(0.95, 0.99)	0.014
Age of wife	0.97	(0.95, 0.99)	0.041
Education			
Tertiary	1	-	
Primary and secondary	2.08	(1.23, 3.51)	0.006
Wife’s education			
Primary and secondary	1	-	
Tertiary	0.62	(0.37, 1.04)	0.068
Employment			
Employed	1	-	
Unemployed	0.92	(0.18, 4.68)	0.924
Wife’s employment			
Employed	1	-	
Unemployed	0.98	(0.58, 1.66)	0.941
Income			
< RM1000	1	-	
RM1001–2000	0.79	(0.38, 1.62)	0.519
RM2001–3000	0.65	(0.30, 1.41)	0.276
>RM 3001	0.56	(0.28, 1.16)	0.117
Number of children			
1–2	1	-	
3–4	0.76	(0.43, 1.37)	0.364
> 5	0.66	(0.31, 1.38)	0.264
Age of youngest child			
Less than 1 year old	1	-	
Between 2 and 5 years old	0.52	(0.26, 1.04)	0.064
More than 5 years old	0.54	(0.28, 1.05)	0.070
Planned pregnancy			
Planned	1	-	
Unplanned	0.81	(0.47, 1.38)	0.435
Wife’s chronic medical illness			
Yes	1	-	
No	0.85	(0.47, 1.53)	0.580

**Table 4:** Associated factors for poor knowledge level by multiple logistic regression

Variable	B^a^	AOR^b^ (95% CI^c^)	*P*-value
Age of respondent	−0.041	0.96 (0.94, 0.99)	0.002
Education			
Tertiary		1	
Primary and secondary	0.960	2.61 (1.49, 4.57)	0.001

Notes:

a Beta coefficient;

b Adjusted odds ratio;

c Confidence interval
